# Patient-Centered Integrated Model of Home Health Care Services in South Korea (PICS-K)

**DOI:** 10.5334/ijic.6576

**Published:** 2023-04-11

**Authors:** Hanbit Mun, Kyunghee Cho, Sanghyun Lee, Youngeun Choi, Seung-Jin Oh, Young-Sung Kim, Migyeung Seo, Ji-Young Park, Serng Bai Pak

**Affiliations:** 1 Department of Family Medicine, National Health Insurance Service Ilsan Hospital, Goyang, Korea; 2 Division of Cardiology, Department of Internal Medicine, National Health Insurance Service Ilsan Hospital, Goyang, Korea; 3 Department of Chronic Disease Management, National Health Insurance Service, Wonju, Korea

**Keywords:** Home health care services, Coordination, Home health care support center, PICS-K, Interdisciplinary team, Integrated care

## Abstract

**Introduction:**

As South Korea is fast becoming an aging society, the need for integrated care of the elderly has increased. ‘Community Integrated Care Initiatives’ have been implemented by the Ministry of Health and Welfare. However, home healthcare is insufficient to meet this need.

**Description:**

The National Health Insurance Service (NHIS) launched the initiative, ‘Patient-Centered Integrated model of Home Health Care Services in South Korea (PICS-K)’. Its purpose is to coordinate home healthcare providers by establishing a home health care support center (HHSC) in public hospitals starting in 2021. The PICS-K has six main features: integration of primary care-hospital-personal care-social services through a consortium, HHSC in hospitals with primary care collaboration, increased accessibility, interdisciplinary team (IDT), patient-centeredness, and education.

**Discussion:**

Integrating healthcare, personal care, and social services at multiple levels is necessary. Accordingly, platforms to share participant information and service records, and institutional payment system reforms are required.

**Conclusion:**

In public hospitals, the HHSC supported primary care, which provides home healthcare. The model combined community healthcare and social services to accomplish the aging-in-place of the homebound population by focusing on their needs. This model will be applicable to other regions in Korea.

## Introduction

### Background

By 2026, Korea is predicted to become a super-aged society [1]. The average number of chronic diseases increases as the population ages; accordingly, medical expenses also increase [2]. In 2020, 12.2% of the elderly in Korea had activities of daily living (ADL)/instrumental activities of daily living (IADL) disabilities, and 18% reported that the reasons for not receiving treatment in hospitals or clinics were related to mobility problems [3]. This increased mobility problems due to aging has made integrated home healthcare in the community necessary for the elderly population.

There are many reasons for investing in maintaining elderly patients in the community instead of hospitals. ‘Post-hospital syndrome’ is one good reason, which comprises rehospitalization, other acute illnesses, and deconditioning observed within 1 month after discharge in elderly patients [4]. In addition, hospital at home appears to offer care that is cost-saving, safe, and more effective than acute hospital admission [5].

Korea has a compulsory health insurance system for all citizens that has been enforced since 1977 and is operated by a single insurer (National Health Insurance Service, NHIS). In addition, since 2008, the NHIS has introduced a long-term care system to care for the elderly who have difficulty performing daily life alone, thereby providing long-term care benefits, such as physical activity or housework support.

In addition, as the population is gradually aging, the Korean Ministry of Health and Welfare selected several regions and carried out the ‘Community Integrated Care Initiatives’ so that residents in need of care can receive community services tailored to their individual needs in the places where they live. [6,7,8]. Home healthcare is one of the key elements of these initiatives. However, nationwide, only 142 medical institutions participated in home healthcare, with a participation rate of 0.4% across all medical institutions. Moreover, the home healthcare service of various medical professionals has been fragmented. Integrated home healthcare services by interdisciplinary teams (such as IDTs of doctors, nurses, and social workers) were only partially carried out [9].

Globally, many studies and guidelines on integrated care for the elderly have been published, laying the foundation for this project [10,11,12,13,14,15]. The World Health Organization (WHO) proposed Integrated Care for Older People (ICOPE) guidelines to establish a health system for a healthy aging population. In 2019, the ICOPE guidelines proposed an extensive integration of long-term care into healthcare systems in three stages. It offers patient-centered integration at the micro-level, cooperative structure among institutions, the integration of healthcare and welfare at the meso-level, and the integration of systems and legislation at the macro-level. The ICOPE integrated model provided the theoretical background for the Patient-Centered Integrated model of Home Health Care Services in South Korea (PICS-K) [10,11].

The PRISMA research project helped frail older adults who lived in the community through the seamless coordination of healthcare and social services [12,13]. The PRISMA project produced improved results (reduced emergency room (ER) visits and the prevalence and incidence of functional decline) at no additional cost [14]. It involves coordinating the services of community-related organizations according to the needs of the elderly as not all services are provided by one institution. Case managers identify customers’ needs, plan the necessary services, connect customers with the services, monitor and coordinate services between providers [15]. The integrated PRISMA model is a practical service model for this initiative.

Home-based primary care (HBPC) in the United States (US) provides direct home-visit medical care, continuous and comprehensive healthcare and social services through IDTs. After HBPC implementation, annual medical expenses and the average length of hospital stay have decreased [16,17,18].

The Program of All-inclusive Care for the Elderly (PACE) in the US provides and coordinates a continuum of medical and social services, including primary care, home healthcare, hospitals, nursing home care, and occupational and recreation therapy. The PACE organizations contributed to cost reduction by delaying nursing home care and shortening hospital stays for frail older adults [19].

However, in South Korea, due to the fee-for-service (FFS) system with low medical cost policy and insufficient multidisciplinary team approach, home medical care and the elderly population care could not be effectively implemented. There is a high demand for an improved essential medical system that is suitable for an aging society. Low out-of-pocket costs owing to the FFS system with extremely low medical cost may be one of the factors that made this initiative possible. Nevertheless, given the soaring rate of Korean national medical expenses relative to gross domestic profit (GDP), it is no longer possible to prepare for the aging era with an FFS system and ever-lasting a low medical cost policy [20].

Hence, community healthcare providers need to be coordinated to provide at-home services with an IDT approach in a patient-centered manner according to the PRISMA and other models. Therefore, the purpose of this initiative was to establish a patient-centered model of home healthcare that provides integrated health and personal care according to the needs of frail older adults by introducing the role of coordination and support centers in public hospitals.

### Problem statement

Goyang City has a total population of 1,079,216 individuals, with 156,861 persons aged ≥65 accounting for 14.5% of the total population, and 16,092 (10.2%) long-term care insurance beneficiaries. There were 20,788 people living with disabilities [1]. Only two patients participated in the primary care home visits, and only five home physician visits were performed in 2020, suggesting that home healthcare, particularly in terms of doctor visits, was essentially non-existent. The average total medical expenses per older adult in Goyang is KRW 3,125,160 per year. However, the total annual medical costs of the most severe grade of long-term care beneficiaries are as high as KRW 17,123,290, which is more than five times higher than that of the general elderly population. The higher the long-term care grade, the higher the annual total medical expenses and hospitalization days [1]. Goyang has eight visiting nursing centers, five primary care home visiting practices, ten disabled family doctors, and eight home nursing centers. Only 0.8% of 578 primary care physician (PCP) offered home visits. Primary care home visits, home nursing, home hospice care, long-term care visiting nurses, visiting bathing, welfare equipment, and various community social services are among the home service provided in the city. Existing home healthcare services are designed to be integrated and individualized for each patient’s needs [21].

## Description of the PICS-K

To achieve ‘aging-in-place’ for homebound patients, the NHIS established an IDT home health care support center (HHSC) in a public hospital in August 2021, launching a PICS-K, which focused on the coordination and support of various community home healthcare services.

### Patient group

The participants were either long-term care beneficiaries, had a disability, or were recipients of home nursing services in Goyang. As of December 2021, 76 people were enrolled, with an average age of 83.58 years old (±10.46), and 75% were women. Table 1 shows the characteristics of the participants of the initiatives. Patients were recruited through promotion materials, consultation, home nursing centers, local providers (PCP and long-term care institutions), or community referrals. Transitional care for managing patients after discharge was also employed for 32 patients referred through the hospital and chronic care was continuously administered after the transitional period. This project is scheduled to be carried out continuously for at least 3 years from 2021.

**Table 1:** The characteristics of patients.


	N	%

Age	83.58 (± 10.46)	

Sex (Women)	57	75%

Long-term care beneficiaries grade		

1 Very heavy need	13	17.1%

2 Moderate need	13	17.1%

3 Partial need	21	27.6%

4 Light need	12	15.8%

5 Alteration in cognition	2	2.6%

Disability grade		

Severely disabled	12	15.8%

Mildly disabled	9	11.8%

Coverage		

National health insurance	62	81.6%

Medical care	13	17.1%

Referral pathway		

Public hospital	32	42.1%

Promotion materials	22	28.9%

Long-term care institution	11	14.5%

Community referrals	11	14.5%


This is a case study of the first year of the initiatives; therefore, the data used were collected only from a short period. The following study will provide data on its impact on patients with consideration of these baseline results.

### The characteristics of PICS-K

#### Process

Patients that were registered at the HHSC underwent five processes: screening, IDT meeting, coordination, monitoring, and counselling. The initial evaluation was the coordinator’s screening, which identified problems in the patient’s condition and home healthcare needs. The IDT meeting produced a patient summary sheet comprising a comprehensive care plan and recommended home services based on the patient information and problem list [Appendix 1]. Home-based services were provided by linking patients to various home providers. The coordinators regularly monitored service fulfillment according to the initial care plans, checked for changes in the patient’s condition, and consulted with patients and providers.

#### Six key features of the model

##### Integrated management: primary care-hospital-personal home care-social service-NHIS

**Figure 1:**
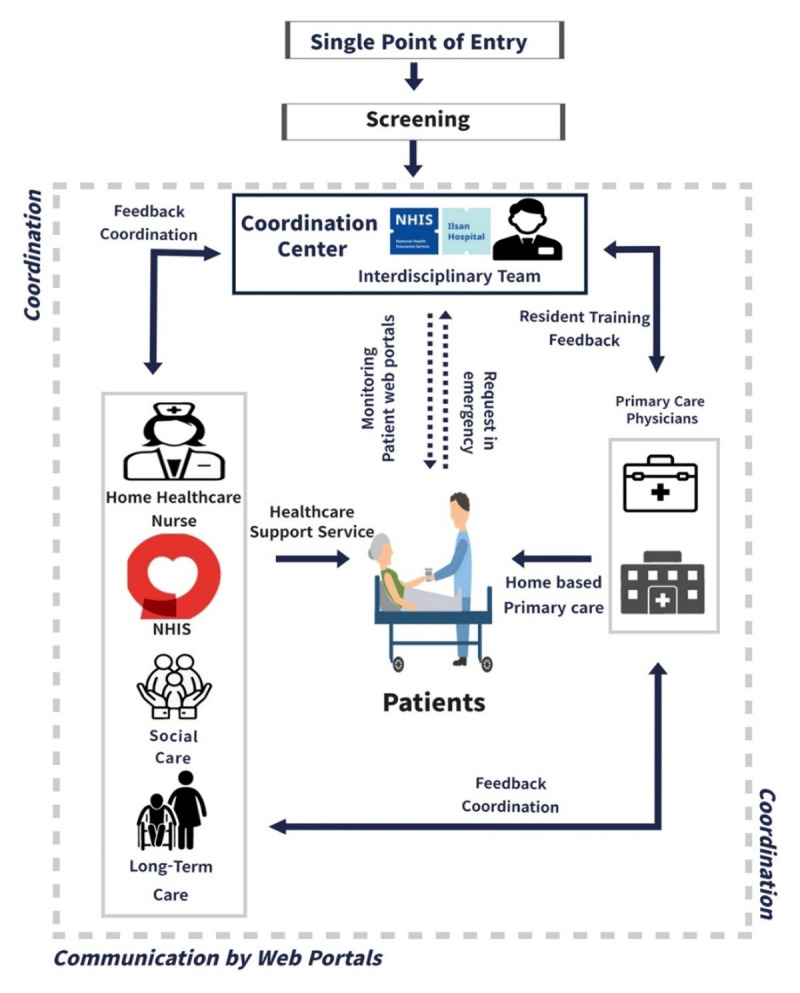
The PICS-K scheme.

In the PICS-K model, integrated management through multiple at-home service providers was performed, focusing on the patient’s needs. It was based on the voluntary participation of community providers and did not require structural merging. Functional and vertical integration between a public hospital and four primary care clinics was achieved. Individual problem lists and care plans were drafted by the IDT based on individual needs and summarised on the participant’s summary sheet [Appendix 1]. This summary was shared with the providers after the participants’ consent. PCP concentrated on regular home visits, whereas the public hospital served as a backup to support primary care with an IDT. Moreover, all existing at-home services were performed according to patients’ needs, including nursing services (home nursing and long-term care visiting nursing), home visit care (long-term care), personal care for the disabled, community social services, and NHIS healthcare services. The NHIS big data were used to discover and recruit patients living with mobility issues in their homes, and information from the NHIS database was used to confirm the diagnosis and medication history of the patients (Figure 1).

##### Public hospital HHSC – Primary care network

**Figure 2:**
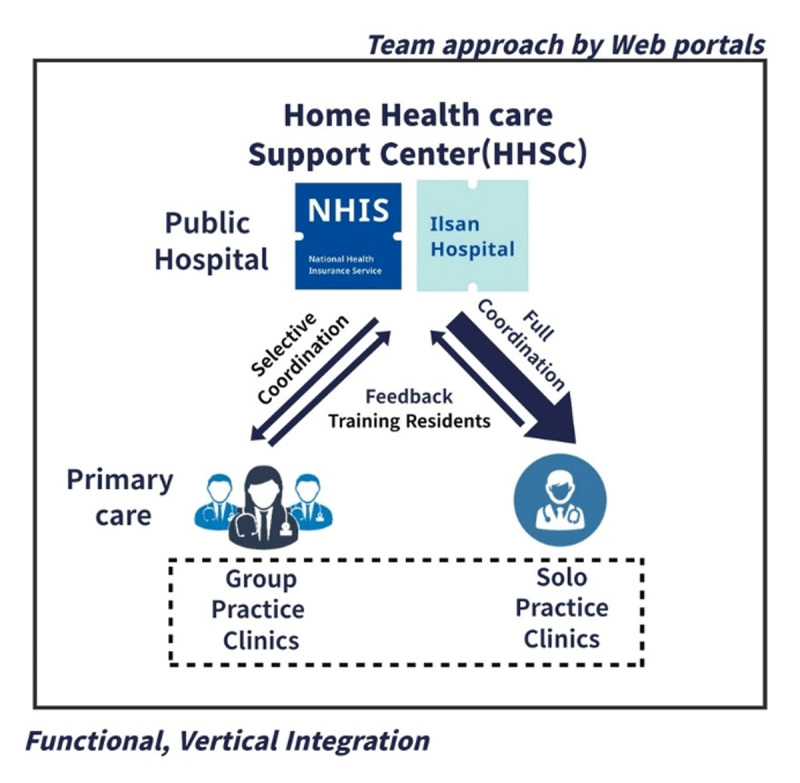
Public hospital – Primary care physician network.

The HHSC played a central role in coordination and was located in a public hospital where the coordinator was stationed. Two coordinators, one full-time and one part-time assistant, were qualified as social workers. The primary approach was a team-based collaboration between public hospitals, community PCP, and long-term care centers. It provides full coordination for solo practitioners and selective coordination for group practitioners who already have team support. The public hospital HHSC delivers the patient’s initial evaluation and summary to the PCP who shares the patient’s condition with the HHSC after the home visit. Even in non-face-to-face patient management, HHSC and PCP function as a team to manage patients together. If the PCP determines a condition to be an emergency, the hospital immediately arranges for an ER visit, hospitalization, and initial intervention. Additionally, for subacute patients discharged from the hospital, the HHSC continued home care and monitoring through referral to a PCP. In this process, the hospital provided two professors of family medicine and two home nursing center nurses to oversee the IDT meeting every day for approximately 30 min (Figure 2).

##### Increased accessibility: Improving time and space restriction

The Kakao channel (a commercial application for customer management) was used by the HHSC and visiting doctors to conduct on-demand consultations with patients and caregivers to increase patient accessibility. Patient and caregiver satisfaction was very high, as consultations on medical, caregiving, and administrative issues could be conducted through the web portals on weekdays during working hours [22]. In addition, periodic wired monitoring was performed for each patient with different cycles depending on the severity of the patient’s condition. The HHSC reviewed and prepared further care plans, depending on the monitoring outcome (whether to continue home health care, ER visits, or hospitalization). A progress report was generated for every patient-related incident, even with simple application counselling and wired monitoring. This progress report was shared among IDT members [23].

If a face-to-face evaluation was warranted or rapid changes in patient’s condition were suspected during counselling, a triage visit for physical examination, blood tests, or medication prescriptions was performed by the center medical staff. Patients and caregivers were instructed first to ask HHSC when their condition changed. Therefore, unnecessary ER visits and hospitalizations could be prevented by the initial call, and ER arrangements were made only when necessary. Thus, as a key function of the HHSC, a triage visit must be included as a primary role of the center.

Furthermore, a discharge conference was convened with the patient, caregiver, inpatient doctor, HHSC doctor, resident, coordinator, at-home nurse, and social worker when the patient was to be discharged from the public hospital and referred to the HHSC. The IDT compiled a list of patient concerns and established medical, nursing, and personal care plans after determining the reason for the patient’s hospitalization, treatment plan, and post-discharge measures. This suggests that suitable at-home healthcare services should be provided. The IDT assisted with the installation of welfare equipment and purchase of relevant supplies if necessary. Patients and caregivers were allowed to attend meetings, and the IDT tried to include their suggestions in the plans as much as possible. The goal was to seamlessly coordinate at-home services after discharge [24].

##### IDT of HHSC: Home-based medical care by coordination

The IDT, comprising family medicine physicians, residents, home nurses, coordinators, and social workers, held IDT meetings daily at the public hospital, and if necessary, PCP or long-term care visiting nurses participated through online meetings [25]. At the IDT meeting, a patient’s summary sheet was drafted based on the patient’s information and screening, and medical, nursing, and personal care plans were confirmed according to the listed problems. The necessary at-home services were determined by the overall needs of the patient and available services. It is essential to designate the exact roles of each player, such as the center, visiting PCP, home nurses, and visiting nurses, and record the services they provide and share. Discussions at the IDT meetings and updated care plans were recorded in the hospital’s electronic medical record (EMR) system.

Moreover, the IDT conference decided on the community provider connections and coordination. The location, patient’s mobility, visit time, patient’s condition and current state, and the availability of home nursing were connection and coordination requirements. Various community resources (welfare centers, disability welfare, rehabilitation, mental health, free equipment support, and NHIS health/nutritional services) were linked based on participants’ needs.

The IDT organized regular consortium meetings comprising the HHSC staffs at the public hospital and other community home-based service providers to establish provider networks and close cooperation. Communication with providers was conducted through the portals and phone, and all related information was recorded in the EMR of the hospital.

##### Patients customized services

Case management is one of the key features of the PICS-K program. In particular, coordinators must be specially trained for this purpose. The coordinators’ duties varied. The coordinator is in charge of the patient’s initial screening and comprehensive evaluation of family relations, main caregiver status, living environment, and medical history. Based on this, a care plan and a patient summary sheet were created. Various home medical and care services were adjusted, such as the visiting medical and nursing services and personal care services, to meet the patient’s needs. Additionally, when necessary, various welfare resources were coordinated in the community. During the first home visit accompanied by the team, the coordinator performed various tasks, such as evaluating the living environment, identifying the needs of the main caregiver, obtaining a consent form for home visits, and guiding patient’s co-payment. In addition, through the Kakao Channel, consultations on patients’ social issues and regular monitoring via wired communication were performed.

We developed a simple and easy screening tool to help coordinators complete screening in less than 5 min. Referring to several existing studies, 12 questions were drawn from five areas: patient information, physical function, cognitive and mental health, nursing treatment, and others [11,26,27,28,29,30]. Patient information included the primary caregiver, location, recent fall history, multi-pharmaceutical use, and social and past histories. The physical function section was mainly evaluated using ADL. Memory loss, behavioral and psychological symptoms of dementia in the previous month, and depressive mood or lethargy in the previous 2 weeks were all assessed for cognitive and mental health. The nursing care section evaluated conditions requiring nursing, including bedsores, the use of nasogastric tubes and foley catheters, and the presence of stomas. The other areas evaluated included the stability of the vital signs, continuous pain, and acute changes in vision or hearing [Appendix 2].

Based on the information gathered, specific problem lists and care plans were prepared. Care plans were categorized into medical, nursing, and personal care plans [30]. The problems were listed in the order of importance. The medical care plan was drafted as precisely as possible by the doctor in the HHSC, nursing plan by the nurse, and personal care plan by the coordinator. Recommendations for suitable services were drawn using algorithms at IDT meetings and coordinated to avoid conflicts between service providers [Appendix 3]. The patient summary sheet was completed at the IDT meeting [Appendix 1].

PCP visited the patients’ homes and cared for them based on the plans listed in the summary sheet. Physical examination, administration of intravenous infusions, nutrient prescriptions, medication reconciliation, and compliance checks were performed. Patient and family education on pressure sore prevention and joint contraction-prevention exercises were conducted. Changes in the patient’s condition and emergency events were also identified. The visit interval was determined based on the patient’s condition [25]. Periodic re-evaluations and monitoring were also performed. Frequently, patient or provider questions were answered through the web portals. When the patient was hospitalized, the coordinator set up a discharge meeting.

##### Education of home health care workforce

As part of the training, family medicine residents accompanied PCP and ‘doctors for the disabled’ and participated in home healthcare. At IDT and discharge meetings, the residents prepared the problem list and initial medical care plans. In addition, they were in charge of answering the questions from the patients through the web portals.

PCP were also educated on home healthcare. In Korea, the concepts of a problem-oriented medical record (POMR) and a team approach are unfamiliar. Therefore, an individualized patient summary was provided to the PCP. It was recommended that each problem in the summary sheet should be addressed at every visit, not just the chief complaint. Gradually, PCP have adopted this perspective [31,32].

### Practical barriers and alternatives

There were four primary barriers aspects: providers, participants, information-sharing platforms, and community welfare. Practical alternatives are proposed here.

#### Providers’ aspect

First, owing to a lack of providers, patients’ waiting list becomes long and the distance between visiting doctors and patients increases. New providers must be recruited through public promotions or related symposiums. Second, communication between the centers and providers was difficult because the providers were unfamiliar with the team approach and were unaware of the importance of coordination. Information-sharing and communication platforms are essential; however, in reality, we communicated with other providers using papers and portals. Despite this limitation, the level of communication tends to improve over time. Third, homebound patients experience various problems and medical conditions. Initially, visiting doctors were not familiar with the process of addressing each issue. However, as the number of visits increased, they gradually became more accustomed to the role of at-home family doctors. The PCP should be requested to access each problem on the list. The problem lists and care plans for homebound patients should be standardized at the level of the academy [29].

#### Patients’ aspects

The IDT recruited homebound patients for whom home healthcare was necessary, and the criteria were relatively broad such as paralysis, terminal disease, bedsores, psychiatric diseases, and cognitive disorders. Standardization of the indications is required for further studies. Specifically, the HHSC should be aware of recently discharged patients who are more likely to be re-hospitalized or visit the ER due to worsening conditions.

#### Information-sharing platform barrier

When an information-sharing platform is lacking, information from other providers cannot be obtained. Therefore, it is often necessary to rely on old medical documents or inaccurate family memory. In the PICS-K study, recent medical and medication histories were secured using the NHIS data. For seamless coordination after the initial connection, it is necessary to check the records of home service providers. The difficulty in follow-up and the need for an efficient team approach tool necessitates an information-sharing platform through the web, tablet or others [23,33].

#### Difficulties in linking to social resources

Most of the participants were not eligible beneficiaries of medical care. For only one tenth of the participants were the disadvantaged and due to the strict qualification criteria for the disabled and basic welfare recipients, it was nearly impossible to obtain help from community welfare organizations or the local government. In Korea, the social welfare budget is increasing; however, the funds were not sufficient to universally provide necessary services, such as packaged lunches and transportation services, and there were additional barriers for homebound patients, such as in-person visits and services at a fixed time. Therefore, to make the best out of what we have right now, it should be mandatory that community social resources be converted into a database, so that they are automatically linked without exception if the qualification criteria are met.

## Legal aspects of barriers and alternatives

The legal aspects of the barriers to visiting doctor and nurses’ initiatives were examined.

### Problems associated with and alternatives to the visiting doctor initiative

#### Problems associated with and alternatives to primary care

Under Korea’s FFS payment system, there are no fees for team performance, such as fees for at-home patient management. Nevertheless, an IDT, which comprises doctors, nurses, social workers, and physical therapists is essential for patient-centered integrated home healthcare.

The PICS-K study proved that HHSC at a public hospital could revitalize community home healthcare by supporting local primary care providers so that a team approach could be activated. Therefore, reformational and institutional change is warranted to promote home healthcare and a team approach, including delivery and payment policy reform, such as risk-adjusted capitation or accountable care organizations (ACOs). Compared with solo practitioners, group practitioners have spare time during the day because they have flexible schedules. Therefore, it is necessary to promote the active participation of group practitioners and formulate policies to encourage group practice. Solo practitioners usually visit at lunchtime or after 6 pm. In addition, to encourage solo practitioners to voluntarily use outpatient times for home healthcare, efficient grouping of patients according to distance is mandatory. Additionally, if nurses and doctors visit is performed together, the effectiveness of home healthcare will increase. Accompanying nurses are naturally included in practices involving home nursing. Nevertheless, in most cases, it is less costly to utilize long-term care visiting nurses instead of home nursing. However, long-term care insurance grouped visiting nursing with personal home care in terms of reimbursement and made them compete for the same funds. Therefore, most bedridden patients and families exhaust their benefits from personal care with no funds left for visiting nurse care. Hence, policy change is required.

#### No incentives for public hospitals

Public hospitals lack incentives because there are no separate coordination fees. It is necessary to set aside separate monthly management fees, such as per-man-per-month (PMPM), or incorporate them into new systems, such as ACOs. In addition, temporary alternatives, such as dispatching coordinators to public hospitals or increasing project budgets to accommodate hired coordinators should be sought.

### Home visit nursing for long-term care

Currently, a daily 4 hours benefits is the maximum allowed under the long-term care system. Thus, personal home care time is always insufficient from the perspective of the primary caregivers. As most personal home care recipients are bedridden patients with deconditioning, the demand for nursing care has increased over time; however, there is a lack of care. Therefore, the utilization of visiting nursing services is very low. In addition, there were no separate fees for procedures performed by visiting nurses. These restrictions cause the underutilization of visiting nursing service in the community. Similar to welfare equipment, benefits due to home-visit nursing service should be separated from that of home-visit care.

## Outcome

Based on the patient satisfaction survey, the overall initiatives of home health care integrated service scored 4.49 out of 5 points and the satisfaction of doctors participating in the initiatives score was 4.14 points (Table 2). The linkage rate of home services increased, and appropriate coordination was made. There was a short-term effect of realizing ‘aging in place’ through end-of-life care at home where seven out of nine severely ill patients living in the community died at home without entering any facility, which warranted long-term research (Table 3). The final results should include changes in medical utilization behavior, such as hospitalization rate, length of hospital stay, and number of ER visits and hospitalizations through the ER. In addition, changes in medical expenses, including hospitalization, outpatient visits, ER use, and total costs will be analyzed for the final result. Analysis of these indicators requires health insurance claims data.

**Table 2:** Satisfaction of patients and providers with the HHSC.


PATIENTS/CAREGIVERS (41)	DOCTORS (7)	COORDINATOR (8)	NURSE (2)

Overall satisfaction with PICS-K (5)	4.49	4.14	5	5

Recommendation (5)	4.88	4.28	4.375	5

Willingness to re-engage (5)	5	4.28	4.375	5


**Table 3:** Service linkage rate and mortality rate during the first four months.


SERVICE LINKAGE RATE (76)			

	PERSON	CASE NUMBER	RATE

Primary care home visits	57	137	75%

Home nursing	36	36	47.4%

Long-term care Home visit nursing	14	14	18.4%

Home visit care	11	11	14.5%

Home visit bathing	3	3	3.9%

Personal care for the disabled	3	3	3.9%

Day-care center	1	1	1.3%

NHIS healthcare services	2	2	2.6%

Home-type hospice	1	1	1.3%

Mortality rate			

	**PERSON**	**RATE**	

Overall mortality	9	11.8%	

Death at home	7	77.8%	


## Sustainability and Spread

The NHIS is considering a home healthcare model for implementation across the country based on the results of its initiatives. Therefore, the model suggested by this project can be applied nationwide. The possible HHSC models are as follows:

### Model 1: NHIS branch model

In this model, a coordinator is deployed to the NHIS regional branch to be oversee patients’ recruitment and link at-home patients to the local community’s PCP. The NHIS seriously considers this model because it is easy to implement and does not require a coordinator to be stationed in a hospital. The essential role of the coordinator is periodic wired monitoring through to manage providers and patients. However, in this model, coordinators sometimes must decide everything independently. Persuading and receiving help from medical institutions in the community can be the greatest obstacle to this model.

### Model 2: Regional public hospital support center model

In this model, coordinators are dispatched to a public hospital and an HHSC is established at the hospital, which makes the HHSC capable of utilizing hospital resources and also makes it easier to support and cope with a participant’s worsening condition. In addition, the transition phase of subacute patients after hospital discharge can be well-managed to prevent the disease from becoming chronic. Still, promoting hospital participation is not easy because there is no fee for patient coordination in the current payment system. However, this model can be used through collaboration with public medical centers or national university hospitals. A hybrid model, in which each center has two coordinators in two different places: the NHIS branch office and the hospital, is under consideration. A system or platform for remote collaboration is required in this model.

## Discussion

The WHO published the ICOPE guidelines for integrated care for the elderly and presented the integration at micro-, meso- and macro-levels as an essential element of personal healthcare. Therefore, this study proposes the integration of many areas, such as home and visiting nursing, health and long-term care insurance data, home healthcare and long-term care committees, and integrated home healthcare fees.

The integrated management of homebound patients requires systematic advancements, such as the provision of a patient information-sharing system and practical tools to enhance coordination. In addition, under the current FFS-based payment system, services other than face-to-face care are not compensated. Thus, it is essential to reform the payment system. In the short term, it is suggested that a new payment model should be created for homebound patients, such as comprehensive management fees PMPM or fees for the nursing procedures of long-term care visiting nurses. Ultimately, institutional reforms are needed to enable voluntary investment in the integrated process, such as the ACO shared-savings program [34,35].

When implementing an ACO, the prerequisites must be fulfilled, such as small size, voluntary participation, and pre-payment method (based on risk-adjusted capitation). The current FFS should be in conjunction with other forms of payment, such as PMPM payment and pay-for-performance (P4P). This kind of payment system can be used to manage at-home and chronically ill patients. The ACO model brings about voluntary delivery system reform because it is coupled with new payment methods, such as shared savings. Collaboration between primary and secondary and tertiary care hospitals within the ACO is essential to reform the delivery system.

## Lessons learned

- For the aging-in-place of homebound patients, the Patient-Centered Integrated model of Home Health Care Services in South Korea (PICS-K)’ initiative was implemented.
- The PICS-K has six main features: integration of primary care-hospital-personal care-social services through a consortium, coordination center in hospitals with primary care collaboration, increased accessibility, interdisciplinary team, patient-centeredness, and education.
- Practically, IDT approaches and home healthcare education are mandatory for healthcare providers to care for homebound patients. Health information technology platforms can facilitate the sharing of patient information and at-home service outcomes among providers and patients.
- Institutionally, it is necessary to use other payment methods, such as comprehensive management fees and PMPM. It is essential to bring voluntary payment and delivery system reform through other mechanisms, such as ACO shared savings programs, to manage patients by a fully integrated interdisciplinary approach.
- Therefore, we propose an IDT-based home health care support center at a public hospital as a model for community integrated home healthcare.

## Conclusion

Under Korea’s current healthcare payment and delivery system, it is difficult to implement home healthcare based on primary care. Korean PCP are unfamiliar with the concept of comprehensive and continuous primary care and the role of IDT-based family physicians. There has been no institutional mechanism to support this type of practice, which is the leading cause of inactive primary care home visit services within national ‘Community Integrated Care Initiatives’.

Therefore, measures to trigger the activation of home healthcare services are as follows:

First, regional hospitals should support that existing community PCP can perform home visits. This was an HHSC model at a public hospital.

Second, support should be given to individuals who agree to build a home healthcare center in the primary care clinic by themselves.

The role of HHSCs may vary depending on the type of PCP. For example, most solo practitioners need full support to participate in a home healthcare program; however, group practitioners who are capable of a team approach, need simple linkage after patients’ recruitment. Thus, it is necessary to adjust the role of the HHSC. However, in reality, most private clinics in Korea are solo practices, and HHSC currently provides full support.

Accordingly, we propose the ‘home health care support center’ model at a public hospital as a home healthcare model in preparation for the nationwide seeding of the Community Integrated Care Initiative in 2026.

## Additional Files

The additional files for this article can be found as follows:

10.5334/ijic.6576.s1 Appendix 1. Example of a patients summary sheet.

10.5334/ijic.6576.s2 Appendix 2. Screening and Assessment.

10.5334/ijic.6576.s3 Appendix 3. Service Algorithm.
