# Supplementary material for: Patient-Centered Integrated Model of Home Health Care Services in South Korea (PICS-K)
Source: Int J Integr Care. 2023 Apr 11;23(2):6. doi: 10.5334/ijic.6576 (PMC10103715; doi:10.5334/ijic.6576)
Supplement: Appendix 1. — Example of a patients summary sheet. [file ijic-23-2-6576-s1.pdf]

Appendix 1. Example of a patients summary sheet

<table>
<tr><th colspan="4">Summary Sheet (Name)</th></tr>
<tr><td>Sex/ Age</td><td colspan="2">Long-term care</td><td>Disability grade</td></tr>
<tr><td>Insurance</td><td colspan="2">Frequented hospital/clinic</td><td>Recruiting path</td></tr>
<tr><td>Resident ID number</td><td colspan="2">Date of Assessment</td><td>Main Care giver</td></tr>
<tr><td colspan="4">Patient’s status</td></tr>
<tr><td colspan="4">Medical progress (Present Illness)</td></tr>
<tr><td colspan="4">Patient’s background</td></tr>
<tr><td colspan="4">- The living environment
- Existing home healthcare service
- The patient’s needs for home healthcare service</td></tr>
<tr><td colspan="2">Medical history</td><td colspan="2">Drug administration history</td></tr>
<tr><td colspan="2">1. Ischemic stroke</td><td colspan="2">Aspirin, Blood pressure medication</td></tr>
<tr><td colspan="2">2. Parkinson's disease</td><td colspan="2">Levodopa and carbidopa</td></tr>
<tr><td colspan="2">3. Type 2 DM</td><td colspan="2">Metformin</td></tr>
<tr><td>Problem list</td><td>Medical plan</td><td>Nursing care plan</td><td>Care plan</td></tr>
<tr><td>Foley catheter status</td><td>Check urine test results once a month</td><td>Foley catheter aseptic replacement
once a month</td><td>The urine bag should always be positioned lower than the bladder</td></tr>
<tr><td>Current service
in use</td><td>Recommended
medical service</td><td>Recommended
nursing care service</td><td>Recommended
care service</td></tr>
<tr><td>Personal home care
(4hours *
5days/week)</td><td>Physicians’ home visit</td><td>Visting Nurse
(Long-term care)</td><td>Home care
(additional once every
two weeks</td></tr>
<tr><td></td><td>Medical payment</td><td>Nursing care
payment</td><td>Care payment</td></tr>
</table>

	₩ 35,400 / a month	₩ 14,400/ a month	
