# Supplementary material for: Patient-Centered Integrated Model of Home Health Care Services in South Korea (PICS-K)
Source: Int J Integr Care. 2023 Apr 11;23(2):6. doi: 10.5334/ijic.6576 (PMC10103715; doi:10.5334/ijic.6576)
Supplement: Appendix 2. — Screening and Assessment. [file ijic-23-2-6576-s2.pdf]

Appendix 2. Screening and Assessment

Screening and Assessment Name:

**1. Social history**		Have you received a long-term care grade?	1. No	2. Yes ( )	
		Have you received a disability grade?	1. No	2. Yes ( )	
		Are you living alone?	1. No	2. Yes	
		Who is your main caregiver?	1.Spouse 2. Parent 3. Children<br>4. Grandchildren 5. Relative 6. Other		
		Do you use a walking aid?	1. No	2. Yes	
		Are you bed-ridden?	1. No	2. Yes	
**2. Past history**		Check illnesses you suffer.	1. None 2. Dementia 3. Stroke<br>4. Parkinson's disease 5. Cancer<br>6. Diabetes 7. Arthritis		
		Draw a circle around only one reason that most directly causes functional decline.	1, 2, 3, 4, 5, 6,<br>7, 8, 9, 10, 11, 12, 13		
		Any surgery for a hip joint, knee joint or ankle joint or fracture surgery within 60 days?	1. No	2. Yes	
		Have you fell in or around your house within six months?	1. No	2. Yes	
		Are you taking more than 10 drugs?	1. No	2. Yes	
		Have you had your guardian receive a prescription in place of you within six months?	1. No	2. Yes	
**3. Physical function**		Can you eat food without others' help?	1. No	2. Yes	
		Can you go to the bathroom without others' help?	1. No	2. Yes	
**Cognitive health**	**4. Cognition**	Do you think your memory is poor to a degree that you have difficulty in everyday life?	1. No	2. Yes	
	**5. Behavior**	Have you ever conducted mentally abnormal behavior for the recent one month?	1. No	2. Yes	
	**6. Depression**	Have you not felt joy and interest in life for two weeks? Do you feel depressed?	1. No	2. Yes	
**Care and treatment**	**7. Bedsore**	Do you have a bedsore?	1. No	2. Yes	
	**8. Nutrition**	Are you subject to L-tube feeding or gastrostomy?	1. No	2. L-tube feeding	3. Adverse events
	**9. Urination Defecation**	Do you urinate or defecate using a foley catheter or artificial anal?	1. No	2. Use a foley catheter or	3. Infection and adverse event

				artificial anal	
**10. Vital sign**		Have you recently experienced unstable blood pressure, pulses, body temperature, or oxygen saturation?	1. stable	2. Nothing special but observed	3. Acute change
**11. Chronic pain**		Do you feel pain that disrupts daily life?	1. No	2. Feel difficulty in daily life	3. Unable to live daily life
**12. Vision/Hearing**		Can't you see or hear almost anything?	1. None	2. Chronic poor Vision/ hearing	3. Acute change in vision/ Hearing
