# Supplementary material for: Patient-Centered Integrated Model of Home Health Care Services in South Korea (PICS-K)
Source: Int J Integr Care. 2023 Apr 11;23(2):6. doi: 10.5334/ijic.6576 (PMC10103715; doi:10.5334/ijic.6576)
Supplement: Appendix 3. — Service Algorithm. [file ijic-23-2-6576-s3.pdf]

Appendix 3. Service Algorithm

	**Screening**			**STEP 2**		
	**Question**		**Ans**	**Assessment**	**Ans**	**Recommended home service**
**1. Social history**	Long-term care grade?			Grade 3 or higher?		Primary care home visit
	Disability grade?			Severely or Mildly?		Physicians for the disabled
	Living alone? Main caregiver					Social Service
	Walking aid?			Type		Durable medical equipment
**2. Past history**	Past history<br>Illness that causes functional decline<br>Cancer			Disease Hx/ terminal cancer		1. Chronic disease management
						2. NHIS health support
						3. Home hospice
	Lower extremity fracture within 60 days?			Fracture area		Home rehabilitation
	Fall within 1 year?					Primary care home visit
	More than 10 drugs?			Drugs list		Polypharmacy management
	Get another person prescription					Primary care home visit
**3. Physical function**	Meal/Using the restroom alone			ADL/ IADL		Primary care home visit
**Cognitive health**	**4. Cognition**	Memory decline?		MMSE		1. Primary care home visit<br>2. Day-care center
	**5. Behavior**	Mentally abnormal behavior		BPSD		Primary care home visit
	**6. Depression**	Lethargy or Depression within 2 weeks?		PHQ-9		Primary care home visit
**Nusing Care treatment**	**7. Bedsore**	Bedsore		Grade, Site		1. Primary care home visit
						2. Visiting/Home nursing
	**8. Nutrition**	L-tube feeding gastrostomy?		Nutritional Assessment		1. Primary care home visit
						2. Visiting/Home nursing
	**9. Urination Defecation**	Foley catheter or artificial anal?				1. Primary care home visit
						2. Visiting/Home nursing

**10. Vital sign**	Recently V/S unstable?				1. Primary care home visit<br>2. Visiting/Home nursing
**11. Pain**	Chronic pain		NRS Score<br>Site		1. Primary care home visit<br>2. Visiting/Home nursing
**12. Vision/ Hearing**	Limited vision/hearing				Physicians for the disabled
	**Recommended healthcare**<br><br>**If All answers of screening are 'No' : Outpatient treatment**<br>**if even one answer is 'No.3' or 'Y' in behavior : Facility entrance**<br>**others belong to home healthcare**<br>**No caregiver or difficulty in being cared : Social care**				
